# Humus Soil Inhibits Antibiotic Resistance Gene Rebound in Swine Manure Composting by Modulating Microecological Niches

**DOI:** 10.3390/microorganisms13030571

**Published:** 2025-03-03

**Authors:** Xiaoxia Hao, Mengting Chen, Weiping Sang, Linyuan Shen, Li Zhu, Dongmei Jiang, Lin Bai

**Affiliations:** 1 Laboratory of Animal Ecology and Environmental Control, College of Animal Science and Technology, Sichuan Agricultural University, Chengdu 611130, China; 14124@sicau.edu.cn (X.H.); jiangdm@sicau.edu.cn (D.J.); 2 State Key Laboratory of Swine and Poultry Breeding Industry, College of Animal Science and Technology, Sichuan Agricultural University, Chengdu 611130, China

**Keywords:** ARGs, animal wastes, composting, microecological niche, host bacteria

## Abstract

Aerobic composting is widely used for the degradation of organic matter, simultaneously reducing the presence of antibiotic resistance genes (ARGs) in swine manure. However, the phenomenon of abundance rebound or even enrichment of ARGs is still a problem. The effect and mechanism of humus soil (Hs) on ARG reduction by adding it into the piles (0% for the control group (CK); 10% for S1 group; 20% for S2 group; and 30% for S3 group) after the thermophilic phase of composting was investigated. The results indicated that Hs promoted organic matter degradation and nitrogen loss. During days 15–36, the greatest reduction of 69.91% in total ARG abundance was observed in S2, while the abundance rebounded by 222.75% in CK and decreased only 13.71% in S3. With the 20% Hs addition, 85.42% abundance reduction for mobile genetic elements (MGEs) and 100% removal rates for *aad*A5, *aad*A9, *sul*1, *sul*2, and *tet*X were achieved. Moreover, the addition of Hs immediately changed the bacterial community structure of the substrate and varied the bacterial community successional direction in the treatments. Additionally, significantly positive correlations (|r| > 0.6; *p* < 0.05) were found between the top 20 genera and ARGs. The potential host bacteria for ARGs changed from *Lactobacillus*, *Fermentimonas*, *Pusillimonas*, and *Ruminofilibacter* in CK to *Lactobacillus*, *Romboutsia*, and *Streptococcus* in S2, highlighting the shift and reduction in host bacteria driven by Hs, which, in turn, influenced the abundance variations in ARGs. This study verified the feasibility of inhibiting the rebound of ARG abundance effectively by influencing the microecological niche in the pile, offering an approach for promoting a reduction in ARGs in animal wastes.

## 1. Introduction

With the rapid development of large-scale and intensive livestock and poultry farming, increasing environmental pollution emerges as a critical concern. Considerable scientific research has focused on antibiotic resistance genes (ARGs) because they are widely spread and pose a significant potential threat to the ecological environment [1,2]. Animal wastes have become a main “reservoir” for antibiotics, antibiotic-resistant bacteria (ARB), and ARGs [3]. According to reports, the abundance of ARGs in animal manures can reach levels as high as 11–13 log copies/g [4]. Furthermore, ARGs in the environment can spread among various microbial communities, including human pathogens, through mechanisms such as conjugation, transduction, and transformation, facilitated by mobile genetic elements (MGEs) [5]. Therefore, it is essential to remove the harmful biological residues in animal manure before they are discharged or reused.

Aerobic composting is commonly employed for the treatment of livestock and poultry manures [6]. In the aerobic composting process, microbial activity propels the decomposition of organic matter, generating substantial heat, which eliminates pathogens and parasite eggs in the manure. Additionally, it facilitates the removal of resistant plasmids and ARGs [7,8,9]. Studies have reported that 21.48–79.63%, 25–78.57%, and 79–97% of ARGs in animal manures (pig, cattle, and birds, respectively) can be removed [10]. However, some ARGs, such as *sul*1, *tet*(33), *tet*G, *aad*A5, and *aad*A9, still accumulated, and their abundance often rebounded in the later stage of composting [11]. Wang et al. even discovered that the total abundance of ARGs did not decrease, but instead, it increased after the composting process [2]. In response to this rebound, researchers have tried to promote the reduction in ARGs by adjusting the optimal physicochemical properties of the raw materials, as well as by incorporating various additives and microbial agents [12,13,14]. Previous reports have indicated that the introduction of substances such as sepiolite, biochar, cauliflower, and woody peat in the raw substrates resulted in a higher reduction rate for both ARGs and MGEs [15,16,17,18]. These studies also have highlighted the crucial role of microbial communities in influencing the variations in ARG abundance throughout the composting process.

Microorganisms in composting systems are always in interaction and competition with each other. Chen et al. reported that the removal efficiency of ARGs during the composting process was enhanced by external heating and the introduction of thermophilic bacteria [19]. The researchers indicated that the reduction in ARG abundance may be attributed to the elevated composting temperature and the introduction of thermophilic bacteria, intensifying the competition of microorganisms for ecological niches in this microecosystem. Moreover, it was found that non-resistant bacteria demonstrate better adaptability to the environment and exhibit higher competitiveness than antibiotic-resistant bacteria (ARB) [20]. It can be inferred that non-resistant bacteria in composting systems are more likely to prevail in competition. As is widely known, humus soils (Hs), found in sparsely populated, minimally polluted high-altitude areas, have little exposure to antibiotics. As a result, they may carry fewer ARGs, with the majority of microbial communities being non-resistant strains. Therefore, it is hypothesized that microbial communities with lower ARGs in humus soils may exhibit greater adaptability and competitiveness in composting systems. They could compete with the original microbial communities in the substrate, occupying more microecological niches. This, in turn, would inhibit the proliferation and dissemination of potential hosts of ARGs, ultimately reducing the overall abundance of ARGs. Reports have shown that adding Hs to cow manure promoted the maturation of the composting product [21], but its effect on ARG reduction is still unclear.

To validate this hypothesis, the swine manure pile underwent artificially rapid cooling immediately after the thermophilic phase of composting. Humus soil (Hs), characterized by a lower abundance of ARGs, was then introduced to assess its impact on the physicochemical properties, dynamic variations in ARGs, and the succession of microbial communities. This study aimed to explore the correlation between ARGs and microbial communities, with a specific focus on how bacterial competition within microecological niches can contribute to the reduction in ARGs. By doing so, this research seeks to provide new insights into waste management and improve the effectiveness of antibiotic resistance gene removal during the composting process. 

## 2. Materials and Methods

### 2.1. Composting Materials

The raw materials used in this study include fresh swine manure obtained from the teaching farm of Sichuan Agricultural University (Ya’an, China), straw purchased from a factory (Suqian, China), and humus soil collected from a mountain in Chongqing city, China. The physicochemical properties of the raw materials are presented in Table 1.

### 2.2. Experimental Design and Sample Collection

Substrates weighing 127 kg were prepared by mixing fresh pig manure and straw in a 3:1 ratio (*w*/*w*, dry weight). Artificial rapid cooling (1 h) was conducted on the 15th day after the thermophilic phase of composting, and the substrates were divided into four piles of equal weight (dry mass). Different proportions (0%, 10%, 20%, and 30% by wet weight) of humus soil were added to each pile, denoted as CK, S1, S2, and S3, and composting continued for 20 days. Intermittent aeration was applied to the four piles at a frequency of 5 min per hour and an airflow rate of 600–800 mL/min. Water was sprayed once a week to maintain a moisture content of 60–75%.

A total of 200 g samples was collected from the upper, middle, and lower layers of the piles on days of 0, 5, 15, 22, 29, and 36 throughout the composting process. Some fresh samples (20 g) were used for the measurement of the moisture content, pH value, and electric conductivity (EC). Another set of samples (100 g) was dried at 65 °C, ground, filtered through a 100-mesh sieve, and stored in a dryer for the determination of total organic carbon (TOC) and total nitrogen (TN). The final set was stored at −80 °C for DNA extraction.

### 2.3. Physicochemical Properties Measurements

The air and pile temperatures were recorded by several thermometers three times per day. The pH and EC values were measured using pH (PHS-320, Century Ark Technology Co., Ltd., Chengdu, China) and EC (DDS-307A, INESA Scientific Instruments Co., Ltd., Shanghai, China) meters, respectively, after the fresh samples were mixed with ultrapure water at a ratio of 1:10 (*w*:*v*) and shaken for 30 min at 200 rpm, followed by 30 min standing. Moisture content was determined by drying the samples at 105 °C for 8 h. TOC, TN, and the germination index (GI) were determined according to the agricultural standard of the People’s Republic of China [22].

### 2.4. Detection of Bacterial Community and ARGs

The bacterial DNA in the substrates was extracted following the instructions of the soil/fecal DNA kit (TGuide S96, Tiangen Biotech Co., Ltd., Beijing, China). The concentration of extracted DNA was measured using a microplate reader (Synergy HTX, Bio-Tek Instruments, Hong Kong, China). The V3-V4 variable regions of the genes were amplified based on the primers 338F (5′-ACTCCTACGGGAGGCAGCA-3′) and 806R (5′-GGACTACHVGGGTWTCTAAT-3′). A 1.8% agarose substrate was used to evaluate the integrity of the amplified fragment. Sequencing libraries were constructed from the purified amplicons, and the qualified libraries were sequenced using Illumina NovaSeq 6000 (Biomarker Technologies, Beijing, China). Clean data were obtained after initial processing, merging, and filtering, and were analyzed for species classification, OTU clustering, and quality analysis.

Real-time quantitative PCR (qRT-PCR) was employed to amplify 74 types of antibiotic resistance genes (ARGs), including aminoglycoside resistance genes (AmRGs), beta-lactam resistance genes (β-LRGs), macrolide-lincosamide-streptogramin B resistance genes (MLSB), multidrug resistance genes (MDR), sulfonamide resistance genes (SRGs), tetracycline resistance genes (TRGs), trimethoprim resistance genes (TrRGs), vancomycin resistance genes (VRGs) and mobile genetic elements (MGEs), as well as the 16S rRNA genes (Table S1). Genes detection was performed using the Wafergen-Smartchip high-throughput fluorescence quantitative PCR system (Microanaly Genetech Co., Ltd., Hefei, China) [23]. Additionally, the melting curves of each PCR product were analyzed to detect the specificity of primer pairs. The abundance of antibiotic resistance genes was expressed as relative abundance (RAs), calculated as follows: RAs of the target gene = 16S rRNA absolute copies × 2^(−ΔCT)^, where ΔCT = CT (target gene) − CT (16S rRNA gene).

### 2.5. Statistical Analysis

All raw data were initially organized and analyzed using Excel 2019 (Microsoft, Atlanta, GA, USA), and the results were presented as mean ± standard deviation (Mean ± SD). One-way analysis of variance (ANOVA) was conducted using SPSS Statistics (v27, IBM, Armonk, NY, USA) to evaluate group differences at a confidence level of 95%. Alpha diversity analysis of microbial communities was performed by Chao1 and Shannon indexes. The similarity of microbial communities was evaluated using Bray–Curtis distance, followed by principal component analysis (PCA). Spearman correlation analysis and network analysis (Gephi 0.10.0) were performed to assess the correlation between bacteria and antibiotic resistance genes.

## 3. Results and Discussion

### 3.1. Effect of Hs on Physicochemical Properties in Composting

**Figure 1:**
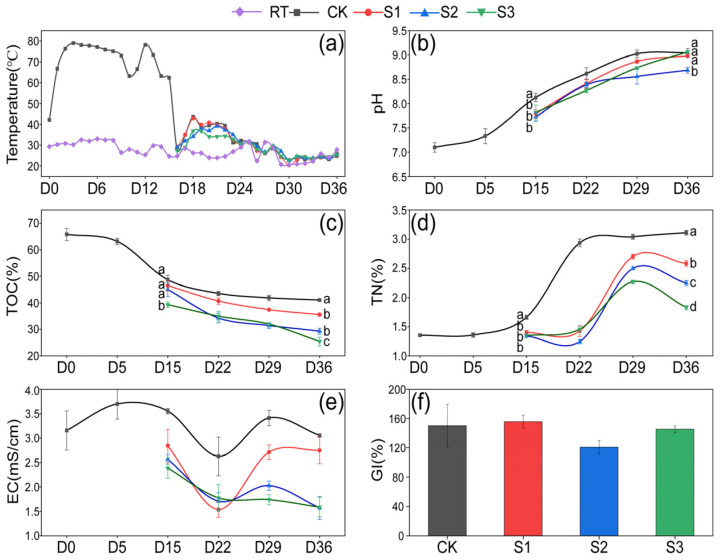
Variation in physical and chemical properties. (**a**) Temperature; (**b**) pH; (**c**) TOC; (**d**) TN; (**e**) EC; (**f**) GI. Letters above the points indicate significant difference between treatments (*p* < 0.05).

The variations in temperature, pH, TOC, TN, EC, and moisture content during composting are illustrated in Figure 1. It can be seen in Figure 1a that the pile temperature rose to 66.8 °C on the first day and remained 63.3–79.0 °C for 14 days during the thermophilic phase. After the addition of additives, transient temperature increases were observed in all treatments on the 18th day, followed by a gradual return to ambient temperature over the next 4–5 days. No significant difference was found between groups. On the 15th day, the pH, TOC, TN, and EC values in the three treatments were observed to be lower than those in CK. This was attributed to the lower initial pH, TOC, and TN values of 7.03, 16.00%, and 0.60% in the humus soil (Table 1), respectively, than in CK. Consequently, lower pH, TOC, and TN were observed during days 15–36 in the treatments. The pH demonstrated a continuous upward trend throughout the composting process, reaching a final range of 8.69–9.06 in all groups (Figure 1b), which was associated with the release of ammonia nitrogen caused by the degradation of organics [24]. TOC decreased by 15.56%, 24.06%, 35.34%, and 38.37%, respectively, in CK, S1, S2, and S3 during days 15–36 (Figure 1c). This decline was observed with an increasing proportion of Hs, likely due to the introduction of numerous microorganisms from the humus soil, enhancing microbial activity and thereby facilitating the decomposition of organic matter [25]. The TN content increased by 87.29%, 84.76%, 67.79%, and 35.98%, respectively, in CK, S1, S2, and S3 from the 15th to the 36th day, with S2 and S3 treatments demonstrating significantly lower (*p* < 0.05) TN values than the CK group (Figure 1d). This indicates an increasing nitrogen loss in composts with a rising proportion of Hs, which is in agreement with Yin’s report that Hs is capable of promoting nitrogen consumption in composting [26].

Throughout the composting process, the EC values in CK ranged from 2.62 mS/cm to 3.48 mS/cm, while those in the treatments varied between 1.45 mS/cm and 2.74 mS/cm (Figure 1e). No significant differences were observed between the groups, all of which remained below 4 mS/cm, suggesting the low phytotoxicity of the composting products [27]. The decrease in EC on day 22 may be linked to microorganisms absorbing salt ions, such as potassium (K⁺) and nitrate (NO₃⁻), for their growth or spore formation [28]. At the end of composting, the GI values were 150.28%, 157.48%, 121.20%, and 142.04% in CK, S1, S2, and S3, respectively (Figure 1f). No statistically significant differences were observed between the groups, but all values met the standard of maturity (GI > 120%) [29]. To sum up, Hs showed a significant influence on the physicochemical properties in S2 and S3. This prompted a direction in subsequent research, focusing on the S2 and S3 groups.

### 3.2. Additive-Induced Antibiotic Resistance Gene Reduction

**Figure 2:**
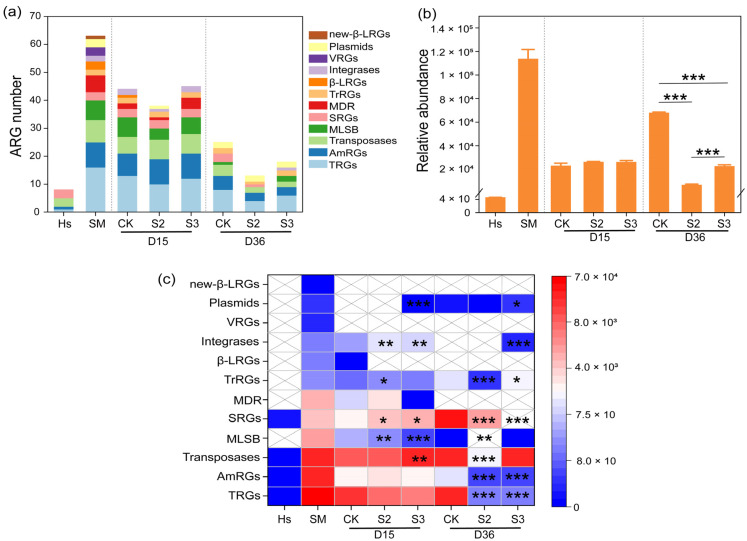
Dynamics of ARGs during composting. (**a**) Gene number; (**b**) gene abundance; (**c**) abundance differences in gene types between CK and S. “*” *p* < 0.05; “**” *p* < 0.01; “***” *p* < 0.001.

**Figure 3:**
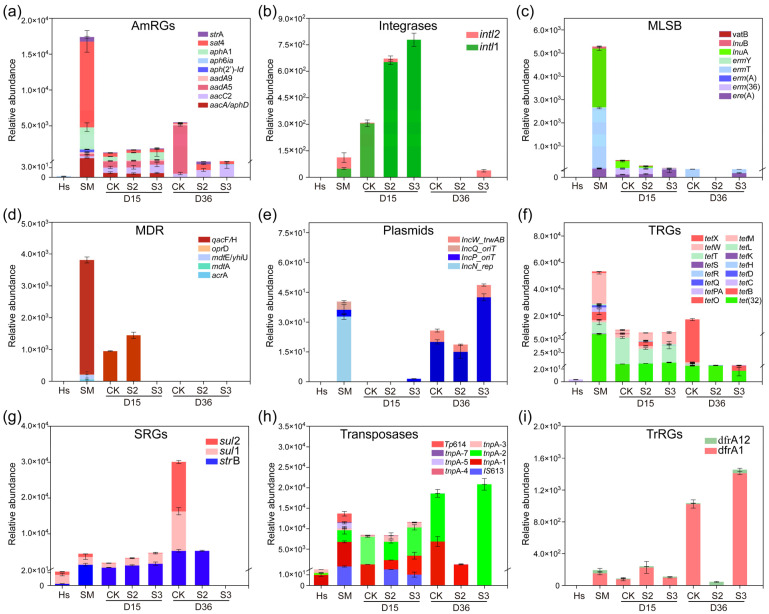
Dynamics of ARG abundance during composting (copy/copies of 16s-rDNA). (**a**) Aminoglycoside resistance genes (AmRGs); (**b**) integrases; (**c**) macrolide-lincosamide-streptogramin B resistance genes (MLSB); (**d**) multidrug resistance genes (MDR); (**e**) plasmids; (**f**) tetracycline resistance genes (TRGs); (**g**) sulfonamide resistance genes (SRGs); (**h**) transposases; (**i**) trimethoprim resistance genes (TrRGs).

The abundance variations in ARGs during the composting process are illustrated in Figure 2 and Figure 3. A total of 66 antibiotic resistance genes were identified in this study, including 9 aminoglycoside resistance genes (AmRGs), 3 beta-Lactam resistance genes (β-LRGs), 8 macrolide-lincosamide-streptogramin B resistance genes (MLSB), 7 multidrug resistance genes (MDR), 1 new beta-lactam resistance genes, 3 sulfonamide resistance genes (SRGs), 16 tetracycline resistance genes (TRGs), 2 trimethoprim resistance genes (TrRGs), and 3 vancomycin resistance genes (VRGs). In addition, 14 mobile genetic elements (MGEs) were detected, including 8 transposases, 2 integrases, and 4 plasmids. Initially, 63 and 8 kinds of ARGs were identified in the substrate and Hs, whereas in the final products of CK, S2, and S3, 25, 13, and 18 gene kinds were detected, respectively (Figure 2a). The introduction of Hs reduced the number of gene kinds. A similar reduction in the number of gene types was reported by Hao et al. [22].

During the 15-day thermophilic phase, the relative abundance of ARGs in the substrate decreased from 9.83 × 10^4^ copies/copy of 16s-rRNA gene to 2.23 × 10^4^ copies/copy of 16s-rRNA gene, marking a 77.27% reduction (Figure 2b). The decline was attributed to the high temperature, which eliminated heat-intolerant microorganisms carrying abundant ARGs [30]. However, the total abundance of ARGs increased by 222.75% in CK during days 15–36, whereas it decreased significantly (*p* < 0.001) by 69.91% and 13.71% in S2 and S3, respectively. A similar abundance rebound in TRGs, AmRGs, transposases, SRGs, and plasmids was observed in CK, whereas reductions were detected in S2 and S3, leading to a significant difference between S and CK (Figure 2c). This rebound of ARG abundance has been reported by Wang et al. [31], and it may be due to the elimination of heat-intolerant microorganisms during the thermophilic phase, creating vacant ecological niches that can be rapidly colonized by bacteria carrying ARGs. Moreover, environmental microorganisms, being the most likely colonizers to enter the pile and occupy niches, are known to carry a substantial amount of ARGs [32]. Following this theory, the decrease in ARG abundance in S2 and S3 could be attributed to the takeover of ecological niches by microbes with lower ARGs (only 4.42 × 10 copies/copy of 16s-rRNA gene) found in humus soil. In comparison to most antibiotic-resistant bacteria (ARB), non-resistant bacteria, or sensitive bacteria, were reported to possess stronger competitiveness and environmental adaptability, inhibiting the proliferation and transmission of ARB [20]. The results suggested that the abundance of ARGs can be significantly reduced by artificially manipulating the occupation of microecological niches in the composting system. Moreover, the addition of Hs, which carries a high abundance of non-resistant bacteria, immediately after the thermophilic phase was proven to be an effective method for ARG reduction.

Specifically, the addition of Hs resulted in a decrease in the majority of detected ARGs. A significant reduction (*p* < 0.05) was observed in the abundance of genes belong to AmRGs, integrases, MLSB, MDR, plasmids, TRGs, transposases, and TrRGs with the addition of 20% Hs in S2 (Figure 3a–f,h,i). The abundance of MGEs rebounded at the end of composting, increasing by 113.47% and 69.29% in CK and S3, respectively, while there was an 85.42% reduction in S2 (Figure 3b,e,h). It has been reported that the reduction in MGEs is a key factor contributing to the decline of ARG abundance during the whole composting process [33], because ARGs can spread and disseminate between different microbial communities through horizontal gene transfer (HGT), primarily mediated by MGEs [34]. In this study, the significant increase in *tnp*A-1, *tnp*A-2, *tnp*A-4, and other MGE abundance in CK and S3 may contribute to the rebound of ARG abundance, whereas the significant reduction in MGE abundance could inhibit the enrichment and rebound of ARGs in S2 (Figure 3h). This provided support for the correlation between ARGs and MGEs.

In particular, during days 15–36, relatively abundance increases of 15227.08%, 189.15%, 804.84%, 45592.46%, and 27753.82% were observed in *aad*A5, *aad*A9, *sul*1, *sul*2, and *tet*X in CK (Figure 3a,f,g). However, they were decreased to undetectable levels in both S2 and S3 treatments, representing a 100% removal. Similar inhibitory effects on the rebound of *sul*1, *aad*A5, *aad*A9, and *tet*X were reported with the addition of shrimp shell powder and diatomite [35,36]. In summary, the addition of 20% humus soil in composting proved to be an effective method for removing ARGs. This prompted a primarily direction in subsequent research, concentrating on the microbial communities and mechanisms of ARG degradation in the S2 treatment.

### 3.3. Successional Changes in Microbial Communities with Hs Addition

**Figure 4:**
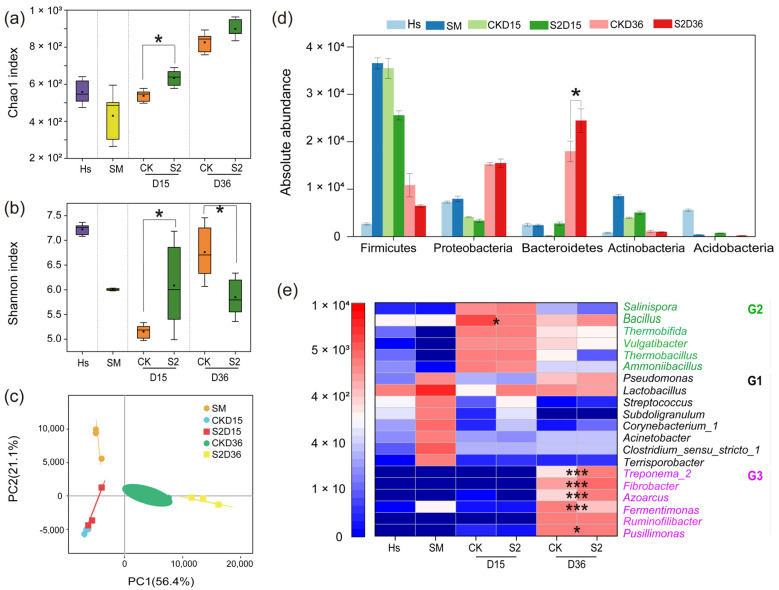
Bacterial community alpha and beta diversities and composition during composting. (**a**) Chao1 index; (**b**) Shannon index; (**c**) principal component analysis (PCA); (**d**) top 5 at the phylum level; (**e**) top 20 at the genus level. “*” *p* < 0.05; “***” *p* < 0.001. Diversity and abundance significant differences between treatments were analyzed by the Mann–Whitney U test and Welch’s *t*-test, respectively. G1, G2, and G3 mean groups 1, 2, and 3.

A total of 5,734,542 high-quality sequences were obtained, clustering into 1234 operational taxonomic units (OTUs) at a 97% similarity level. The Chao1 index increased with composting, and the higher values in S2 than in CK on both the 15th and 36th days were attributed to the original higher species richness in Hs than in CK (Figure 4a). The Shannon index increased with composting in CK, but lower (*p* < 0.05) values were observed in S2 than in CK on the 36th day (Figure 4b). This result may be due to the intensive bacterial competition in S2 caused by the Hs, inhibiting some of the bacteria and leading to the diversity reduction in microbial communities. Principal component analysis (PCA) based on Bray–Curtis dissimilarity was conducted to evaluate bacterial beta diversity between groups (Figure 4c). All the samples were clustered into different groups between CK and S2 on both the 15th and 36th days, illustrating significant different bacterial diversity amended by Hs.

The bacterial community abundance is shown in Figure 4d,e. At the phylum level, Firmicutes, Proteobacteria, Bacteroidetes, and Actinobacteria accounted for 82.80–93.70% of the total sequences in all groups (Figure 4d), which was in accordance with previous reports [37,38]. During days 15–36, the abundance of Firmicutes and Actinobacteria decreased while Proteobacteria and Bacteroidetes increased to become the dominant phyla. At the end of composting, a significantly higher (*p* < 0.05) abundance of Bacteroidetes was observed in S2 (2.44 × 10^4^ OTUs) than that in CK (1.80 × 10^4^ OTUs), indicating that the addition of Hs promoted the proliferation of Bacteroidetes, which is specialized in degrading cellulose and chitin [39].

At the genus level, the initially dominant group G1 including *Pseudomonas*, *Lactobacillus*, *Acinetobacter*, *Streptococcus*, *Subdoligranulum*, *Corynebacterium*_1, *Clostridium*_*sensu*_*stricto*_1, and *Terrisporobacter*, accounted for 53.22% of the total sequences in swine manure (Figure 4e). However, bacterial group G2, including *Bacillus*, *Thermobifida*, *Vulgatibacter*, *Thermobacillus*, and *Ammoniibacillus*, occupied the main position after composting for 15 days in CK, while *Salinispora*, *Vulgatibacter*, and *Thermobacillus* were the top three genera in S2 following Hs addition. It can be observed that the addition of Hs immediately changed the bacterial community structure of the materials, with a similar variation observed in Figure 4d. The result was attributed to the greatly different dominant bacterial community in Hs (Figure S1). The main bacteria were stabilized to *Fermentimonas*, *Ruminofilibacter*, and *Pusillimonas* in CK, and *Treponema*_2, *Fibrobacter*, *Azoarcus*, and *Ruminofilibacter* in S2 at the end. This significantly different bacterial community succession varied by Hs was owing to the effect of its introduced microbiota on the competitiveness of indigenous bacteria in the microecosystem [19].

Moreover, a significantly lower abundance of *Bacillus* was observed in S2 than in CK. It was reported the *Bacillus subtilis* secretes bacillomycin, which exerts inhibitory effects on pathogenic bacteria such as *Salmonella*, *Escherichia coli*, *Bacillus cereus*, and *Listeria*, associated with foodborne illnesses [40]. In addition, a higher abundance of *Fibrobacter*, *Azoarcus*, and *Treponema*_2 and a lower abundance of *Pusillimonas* and *Fermentimonas* were observed in S2 on the 36th day (*p* < 0.05). *Fibrobacter*, a key cellulose-degrading bacterium, plays a direct role in maintaining the ecological balance of the gut microbiota and internal equilibrium [41]. *Azoarcus*, an endophytic nitrogen-fixing bacterium, primarily participates in biological nitrogen fixation processes, contributing to nitrogen balance in soil and ecosystems [42]. *Treponema*_2 is also one of the cellulolytic bacteria [43]. *Pusillimonas* is a typical denitrifying bacterium that drives N_2_O emissions and occurs primarily during compost maturation [44]. *Fermentimonas* are mainly present in the anaerobic fermentation process and can promote the hydrolysis of swine manure [45]. The results of this study suggest that the addition of Hs right after the thermophilic phase during composting changed the bacterial structure in the compost and was beneficial to the growth of cells which are good at degrading cellulose and minimizing nitrogen loss.

### 3.4. Degradation Mechanism of Antibiotic Resistance Genes (ARGs)

**Figure 5:**
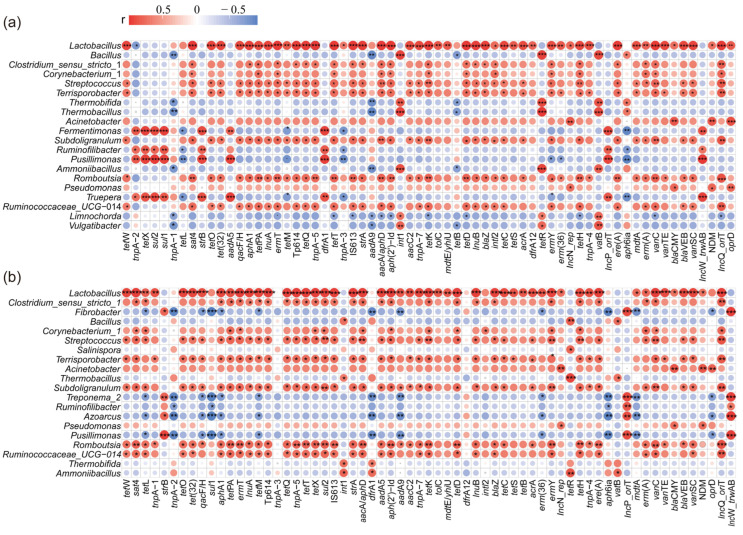
Heatmap of Spearman’s correlation coefficients between the ARGs and the bacterial community (top 20 genera). (**a**) CK; (**b**) S2. Red spots represent positive correlations between genes and genera, blue spots represent negative correlations between genes and genera, and the size of spot represents the value of the correlation coefficient. “*” *p* < 0.05; “**” *p* < 0.01; “***” *p* < 0.001.

**Figure 6:**
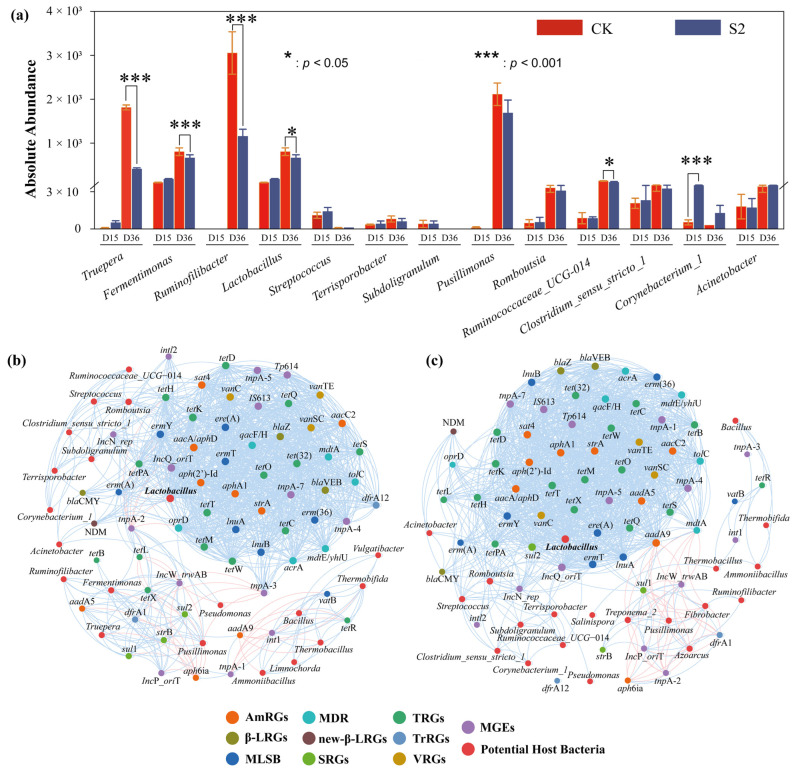
Co-occurrence network analysis of potential host bacteria of ARGs in (**a**) CK and (**b**) S2. (**c**) Variations in potential host bacteria. Nodes in different colors correspond to specific ARGs or different bacteria, blue edges represent positive correlations between specific nodes, red edges represent negative correlations between specific nodes, and the thickness of the line represents the value of the correlation coefficient.

To understand the relationship between the bacterial community and ARGs, Spearman correlation analysis was performed with the top 20 genera and all ARGs in both CK and S2 (Figure 5). Subsequently, a co-occurrence network analysis based on Spearman correlation coefficients was conducted to reveal the potential host bacteria for ARGs and provide insights into the mechanisms underlying ARG degradation during composting (Figure 6). The results showed that the top 20 genera were significantly positively (|r| > 0.6, *p* < 0.05) correlated with 58 ARGs, and a significant negative (|r| > 0.6, *p* < 0.05) correlation was observed with 5 ARGs in CK (Figure 5a). In the S2 treatment, bacteria displayed a significant positive (|r| > 0.6, *p* < 0.05) correlation with 54 ARGs and a negative (|r| > 0.6, *p* < 0.05) correlation with 6 ARGs (Figure 5b). These findings suggested a clear correlation between variations in ARG abundance and the potential host bacteria. For example, *Lactobacillus* was observed to be positively correlated (*p* < 0.05) with 48 and 51 ARGs in CK and S2, which marked *Lactobacillus* as a potential host for most ARGs in this study.

Specifically, strong positive correlations (|r| > 0.6, *p* < 0.05) between specific types of ARGs (AmRGs, TRGs, SRGs, and MLSB) and bacteria such as *Lactobacillus*, *Fermentimonas*, *Pusillimonas*, and *Ruminofilibacter* were observed in CK, suggesting that these bacteria could serve as potential hosts for the four types of ARGs. However, the same four types of ARGs were positively related (|r| > 0.6, *p* < 0.05) to the bacteria *Lactobacillus*, *Romboutsia*, and *Streptococcus* in S2, identifying them as the potential hosts. The major potential host bacteria changed with the addition of Hs, which may result in variations in ARG abundance. As is shown in Figure 5 and Figure 6a, the strongest correlated potential hosts *Fermentimonas*, *Pusillimonas*, and *Ruminofilibacter* in CK remained at a high abundance at the end of composting, while the most correlated bacteria *Romboutsia* and *Streptococcus* in S2 were at lower levels. Additionally, a significantly lower abundance of main potential host genera except for *Corynebacterium*_1 was detected in S2 (Figure 6a). For instance, the abundance of genes including AmRGs, TRGs, SRGs, and MLSB was 4.88 × 10, 1.52 × 10^2^, 5.24 × 10^3^, and 0 copies/copy of 16s-rRNA gene in S2, respectively, while they were much more abundant in CK (5.46 × 10^3^, 1.69 × 10^4^, 3.01 × 10^4^, and 1.07 × 10 copies/copy of 16s-rRNA gene). The fate of genes has been proven to be connected with their hosts by Liu et al. [46].

Additionally, the potential host bacteria for *sul*1, *sul*2, *aad*A5, *aad*A9, and *tet*X in CK were identified as *Fermentimonas*, *Ruminofilibacter*, *Pusillimonas*, and *Truepera*, with a total abundance increase of 189.15% to 45592.46% during days 15–36. However, a significant negative correlation (|r| > 0.6; *p* < 0.05) with bacteria *Ruminofilibacter* and *Pusillimonas* and genes *sul*1 and *sul*2 was found in S2. The potential host bacteria for *sul*1, *sul*2, *aad*A5, *aad*A9, and *tet*X turned to be *Lactobacillus*, *Romboutsia*, *Streptococcus*, and *Subdoligranulum*, resulting in the complete removal (100%) of these genes. Similar findings were reported by Sun et al. [47], who indicated that changes in the succession of potential host bacteria had a greater impact on the spread of ARGs. These results highlight the role of humus soil in reducing ARGs and their hosts, which are less competitive [20], leading to less microecological niche occupation in the compost.

Network analysis further revealed a significant positive correlation (|r| > 0.6; *p* < 0.05) between mobile genetic elements (MGEs) and 40 ARGs in both CK and S2 groups (Figure 6b,c). This suggested that horizontal gene transfer mediated by MGEs might play a crucial role in the variation in ARG abundance during composting, as supported by previous research [33]. In this study, both the total ARGs and MGEs were significantly rebounded in CK, with abundance increases of 222.75% and 113.47% during the final 20 days, respectively (Figure 3). However, they were reduced by 69.91% and 85.42% in S2, indicating a lower frequency of MGE-mediated horizontal transfer events, thereby inhibiting the enrichment and rebound of ARGs through Hs amendment. Furthermore, the co-occurrence analysis also showed a concentrated positive relationship among genes in both CK and S2, with gene connections in S2 being enhanced, indicating that Hs promoted the reduction in total ARGs by removing some connected genes except for MGEs.

**Figure 7:**
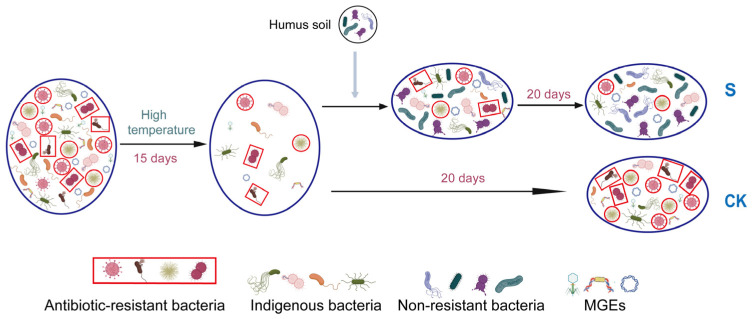
Schematic diagram of the mechanism of ARG reduction during aerobic composting. The different shapes and colors in the bacteria represent distinct bacterial communities. The structural changes between these communities reflect shifts in the occupancy of microecological niches within the composting microecosystem.

In summary, this study provides insights into the effect of Hs on ARG rebound. From the perspective of connections among bacteria and genes in this composting microecosystem, the abundance reduction in the potential host bacteria, the changes in major potential host bacteria, the horizontal gene transfer mediated by MGEs, and the positive relationship among genes and bacteria all contributed to ARG reduction. During this process, the microecological niche occupation caused by Hs addition played a key role in triggering ARG rebound control by using the connection among bacteria and genes during the later stage of composting. The potential process of microecological niche occupation and its effect on ARGs are illustrated in Figure 7 to explain the mechanisms.

## 4. Conclusions

Humus soil (Hs) with a lower ARG abundance proved effective in inhibiting the rebound or enrichment of antibiotic resistance genes during swine manure composting. The results revealed that Hs facilitated the reduction in the total ARG and MGE abundance, with specific genes such as *aad*A5, *aad*A9, *sul*1, *sul*2, and *tet*X being completely removed. With 20% Hs addition, an immediately changed bacterial community structure was observed in the substrate, while a significantly varied bacterial community successional direction was obtained. The main potential host bacteria for ARGs changed from *Lactobacillus*, *Fermentimonas*, *Pusillimonas*, and *Ruminofilibacter* to *Lactobacillus*, *Romboutsia*, and *Streptococcus*. It can be concluded from all the results that the rebound of ARG abundance was effectively inhibited by the addition of Hs which carried rich non-host bacteria that can adjust the microecological niche occupation situation in the composting system and change the bacterial community structure and dominant potential hosts of ARGs. However, the efficacy of Hs was not consistent across all treatments, and more studies are needed in the future to investigate the specific conditions for effective microecological niche occupation on ARG reduction.

## Figures and Tables

**Table 1:** Physicochemical properties of raw materials.

Materials	Moisture (%)	TOC (%)	TN (%)	pH Value
Swine manure (SM)	73.55 ± 0.01	66.57 ± 1.93	1.75 ± 0.07	6.83 ± 0.21
Straw	14.86 ± 0.02	71.82 ± 1.85	0.56 ± 0.02	5.24 ± 0.24
Humus soil (Hs)	43.53 ± 0.01	16.00 ± 1.53	0.60 ± 0.03	7.03 ± 0.01

## Data Availability

Data will be made available on request.

## Supplementary Materials

The following supporting information can be downloaded at: https://www.mdpi.com/article/10.3390/microorganisms13030571/s1, Figure S1: The top 20 genera in humus soil; Table S1: The selected primer sets for target genes.
