# Supplementary material for: Humus Soil Inhibits Antibiotic Resistance Gene Rebound in Swine Manure Composting by Modulating Microecological Niches
Source: Microorganisms. 2025 Mar 3;13(3):571. doi: 10.3390/microorganisms13030571 (PMC11944299; doi:10.3390/microorganisms13030571)
Supplement: Supplementary file 1 [file microorganisms-13-00571-s001.zip › microorganisms-3472045-supplementary.pdf]

# Supplementary Materials

**Humus soil inhibits antibiotic resistance genes rebound in swine manure composting by modulating microecological niches**

Xiaoxia Hao[a,b], Mengting Chen[a,b], Weiping Sang[a,b], Linyuan Shen[b], Li Zhu[b], Dongmei Jiang[a], Lin Bai[a,b*]

Xiaoxia Hao and Mengting Chen contributed equally to this work.

*Corresponding author.

Postal address: Huimin Road 211, Chengdu, 611130, Sichuan, China.

E-mail address: 10384@sicau.edu.cn

[a] *Lab of Animal Ecology and Environmental Control, College of Animal Science and Technology, Sichuan Agricultural University, Chengdu, 611130, P. R. China.*

[b] *State Key Laboratory of Swine and Poultry Breeding Industry，College of Animal Science and Technology, Sichuan Agricultural University, P. R. China.*

**Table S1**

The selected primer sets for target genes.

| Gene Name | Forward Primer | Reverse Primer | Classification |
|---|---|---|---|
| aacC2 | ACGGCATTCTCGATTGCTTT | CCGAGCTTCACGTAAGCATTT | Aminoglycoside |
| aacA/aphD | AGAGCCTTGGGAAGATGAAGTTT | TTGATCCATACCATAGACTATCTCATCA | Aminoglycoside |
| aac(6')-II | CGACCCGACTCCGAACAA | GCACGAATCCTGCCTTCTCA | Aminoglycoside |
| sat4 | GAATGGGCAAAGCATAAAAACTTG | CCGATTTTGAAACCACAATTATGATA | Aminoglycoside |
| aac(6')-Ib | CGTCGCCGAGCAACTTG | CGGTACCTTGCCTCTCAAACC | Aminoglycoside |
| aadA2-1 | ACGGCTCCGCAGTGGAT | GGCCACAGTAACCAACAAATCA | Aminoglycoside |
| aadA5 | ATCACGATCTTGCGATTTTGCT | CTGCGGATGGGCCTAGAAG | Aminoglycoside |
| aph(2')-Id | TGAGCAGTATCATAAGTTGAGTGAAAAG | GACAGAACAATCAATCTCTATGGAATG | Aminoglycoside |
| aadD | CCGACAACATTTCTACCATCCTT | ACCGAAGCGCTCGTCGTATA | Aminoglycoside |
| aadA2-2 | CAATGACATTCTTGCGGGTATC | GACCTACCAAGGCAACGCTATG | Aminoglycoside |
| aadA9 | CGCGGCAAGCCTATCTTG | CAAATCAGCGACCGCAGACT | Aminoglycoside |
| aphA1 | TGAACAAGTCTGGAAAGAAATGCA | CCTATTAATTTCCCCTCGTCAAAAA | Aminoglycoside |
| aadE | TACCTTATTGCCCTTGGAAGAGTTA | GGAACTATGTCCCTTTTAATTCTACAATCT | Aminoglycoside |
| str | AATGAGTTTTGGAGTGTCTCAACGTA | AATCAAAACCCCTATTAAAGCCAAT | Aminoglycoside |
| strA | CCGGTGGCATTTGAGAAAAA | GTGGCTCAACCTGCGAAAAG | Aminoglycoside |
| spcN | GCTATGTGCTGGTGGACTGG | GGAACCACTCGACGAACTCG | Aminoglycoside |
| aadA10 | ACAGGCACTCAACGTCATCG | CGCGGAGAACTCTGCTTTGA | Aminoglycoside |
| aac(6')I1 | GGGAATTATCGGAATAGCTCTTGG | TTGGGCTGTTCTTCCTAGCTAA | Aminoglycoside |
| aac(6')-Iy | GCCTCAATCCGCCACGATTA | ACGCGCTCTGTTTCCTCAAA | Aminoglycoside |
| aph6ia | CGCTGGGAGCTGAAGAGG | AGCATCGTGCTGCTCTCC | Aminoglycoside |

| | | | |
|---|---|---|---|
| blaACC-1 | CACACAGCTGATGGCTTATCTAAAA | AATAAACGCGATGGGTTCCA | Beta Lactam |
| blaMOX/blaCMY | CTATGTCAATGTGCCGAAGCA | GGCTTGTCCTCTTTCGAATAGC | Beta Lactam |
| blaPAO/PDC | CGCCGTACAACCGGTGAT | GAAGTAATGCGGTTCTCCTTTCA | Beta Lactam |
| blaVEB | CCCGATGCAAAGCGTTATG | GAAAGATTCCCTTTATCTATCTCAGACAA | Beta Lactam |
| bla1 | GCAAGTTGAAGCGAAAGAAAAGA | TACCAGTATCAATCGCATATACACCTAA | Beta Lactam |
| blaROB | GCAAAGGCATGACGATTGC | CGCGCTGTTGTCGCTAAA | Beta Lactam |
| blaOXY-2 | CGTTCAGGCGGCAGGTT | GCCGCGATATAAGATTTGAGAATT | Beta Lactam |
| blaPSE | TTGTGACCTATTCCCCTGTAATAGAA | TGCGAAGCACGCATCATC | Beta Lactam |
| blaCMY | AAAGCCTCATGGGTGCATAAA | ATAGCTTTTGTTTGCCAGCATCA | Beta Lactam |
| blaTLA | ACACTTTGCCATTGCTGTTTATGT | TGCAAATTTCGGCAATAATCTTT | Beta Lactam |
| blaZ | GGAGATAAAGTAACAAATCCAGTTAGATATGA | TGCTTAATTTTCCATTTGCGATAAG | Beta Lactam |
| blaVIM | GCACTTCTCGCGGAGATTG | CGACGGTGATGCGTACGTT | Beta Lactam |
| blaCTX-M | GCGATAACGTGGCGATGAAT | GTCGAGACGGAACGTTTCGT | Beta Lactam |
| NDM | GGCCACACCAGTGACAATATCA | CAGGCAGCCACCAAAAGC | new Beta Lactam |
| blaCTX-M-1,3,15 | CGTACCGAGCCGACGTTAA | CAACCCAGGAAGCAGGCA | Beta Lactam |
| blaPER | GCAAATGAAGCGCAGATGC | GACCACAGTACCAGCTGGTA | Beta Lactam |
| blaOXY-1 | AAAGGTGACCGCATTCGC | CCAGCGTCAGCTTGCG | Beta Lactam |
| qnrA | AGGATTTCTCACGCCAGGATT | CCGCTTTCAATGAAACTGCAA | Fluoroquinolone |
| ISCR1 | ATGGTTTCATGCGGGTT | CTGAGGGTGTGAGCGAG | Insertional sequence |
| int1 | GCCTTGATGTTACCCGAGAG | GATCGGTCGAATGCGTGT | Integrase |
| intl2 | TGCTTTTCCCACCCTTACC | GACGGCTACCCTCTGTTATCTC | Integrase |

| | | | |
|---|---|---|---|
| intl3 | GCCACCACTTGTTTGAGGA | GGATGTCTGTGCCTGCTTG | Integrase |
| erm(36) | GGCGGACCGACTTGCAT | TCTGCGTTGACGACGGTTAC | MLSB |
| ermT | GTTCACTAGCACTATTTTTAATGACAGAAGT | GAAGGGTGTCTTTTTAATACAATTAACGA | MLSB |
| ermX | GCTCAGTGGTCCCCATGGT | ATCCCCCCGTCAACGTTT | MLSB |
| lnuA | TGACGCTCAACACACTCAAAAA | TTCATGCTTAAGTTCCATACGTGAA | MLSB |
| vat(E) | GACCGTCCTACCAGGCGTAA | TTGGATTGCCACCGACAATT | MLSB |
| ermY | TTGTCTTTGAAAGTGAAGCAACAGT | TAACGCTAGAGAACGATTTGTATTGAG | MLSB |
| carB | GGAGTGAGGCTGACCGTAGAAG | ATCGGCGAAACGCACAAA | MLSB |
| ere(A) | GATAATTCTGCTGGCGCACA | GCAGGCGTGGTCACAAC | MLSB |
| ere(B) | TCGTATATGGCGGGCGTAGTA | GGTCCAAGATGGGTGAATGCA | MLSB |
| erm(A) | TCGTTGAGAAGGGATTTGCGA | TTGCATGCTTCAAAGCCTGTC | MLSB |
| erm(B) | GAACACTAGGGTTGTTCTTGCA | CTGGAACATCTGTGGTATGGC | MLSB |
| lnuB | GGATCGTTTACCAAAGGAGAAGG | AGCATAGCCTTCGTATCAGGAA | MLSB |
| erm(34) | AAAGCGGTTTACAAGCGTTTCG | GGGTGCTCTAGGGTTGTTTAGTG | MLSB |
| erm(35) | CCTTCAGTCAGAACCGGCAA | GCTGATTTGACAGTTGGTGGTG | MLSB |
| vatB | GCAATTGTTGCTGCGAATTCAG | GTGCTGACCAATCCCACCA | MLSB |
| lnuC | GGGTGTAGATGCTCTTCTTGGA | CTTTACCCGAAAGAGTTTCTACCG | MLSB |
| acrB | AGTCGGTGTTCGCCGTTAAC | CAAGGAAACGAACGCAATACC | Multidrug |
| acrF | GCGGCCAGGCACAAAA | TACGCTCTTCCCACGGTTTC | Multidrug |
| adeA | CAGTTCGAGCGCCTATTTCTG | CGCCCTGACCGACCAAT | Multidrug |
| cmr | CGGCATCGTCAGTGGAATT | CGGTTCCGAAAAAGATGGAA | Multidrug |

| | | | |
|---|---|---|---|
| acrA | GGTCTATCACCCTACGCGCTATC | GCGCGCACGAACATACC | Multidrug |
| tetU | GTGGCAAAGCAACGGATTG | TGCGGGCTTGCAAAACTATC | Multidrug |
| mdtE/yhiU | CGTCGGCGCACTCGTT | TCCAGACGTTGTACGGTAACCA | Multidrug |
| mexA | AGGACAACGCTATGCAACGAA | CCGGAAAGGGCCGAAAT | Multidrug |
| acrR | GCGCTGGAGACACGACAAC | GCCTTGCTGCGAGAACAAA | Multidrug |
| qacF/H | TCGCAACATCCGCATTAAAA | ATGGATTTCAGAACCAGAGAAAGAAA | Multidrug |
| cmlA1 | TAGGAAGCATCGGAACGTTGAT | CAGACCGAGCACGACTGTTG | Multidrug |
| cmx(A) | GCGATCGCCATCCTCTGT | TCGACACGGAGCCTTGGT | Multidrug |
| nimE | TGCGCCAAGATAGGGCATA | GTCGTGAATTCGGCAGGTTTA | Multidrug |
| emrB/qacA | CTTTTCTCTAACCGTACATTATCTACGATAAA | AGAACGTAGCGACTGATAAAATGCT | Multidrug |
| oprD | ATGAAGTGGAGCGCCATTG | GGCCACGGCGAACTGA | Multidrug |
| ttgA | ACGCCAATGCCAAACGATT | GTCACGGCGCAGCTTGA | Multidrug |
| ttgB | TCGCCCTGGATGTACACCTT | ACCATTGCCGACATCAACAAC | Multidrug |
| mexE | GGTCAGCACCGACAAGGTCTAC | AGCTCGACGTACTTGAGGAACAC | Multidrug |
| mtrD | CGGAGTCCATCGACCATTTG | ATCGTCGGCAAGGAGAATCA | Multidrug |
| tolC | GGCCGAGAACCTGATGCA | AGACTTACGCAATTCCGGGTTA | Multidrug |
| marR | GCTGTTGATGACATTGCTCACA | CGGCGTACTGGTGAAGCTAAC | Multidrug |
| floR | AACCCGCCCTCTGGATCA | GCCGTCGAGAAGAAGACGAA | Multidrug |
| mdtA | ACAAGCCCAGGGCCAAC | CCTTAATGGTGCCTTCGGTTTC | Multidrug |
| qacA/B | AAGGGCCACTGCATTAGCTG | CCAGTCCAATCATGCCTGCA | Multidrug |
| catB3 | GCACTCGATGCCTTCCAAAA | AGAGCCGATCCAAACGTCAT | Others |

| | | | |
|---|---|---|---|
| catB8 | CACTCGACGCCTTCCAAAG | CCGAGCCTATCCAGACATCATT | Others |
| catA1 | GGGTGAGTTTCACCAGTTTTGATT | CACCTTGTCGCCTTGCGTATA | Others |
| bacA | ATCCGCGGCACCCTGA | CCTGCTTGATGGACTTGATGAAGA | Others |
| IncN_korA | GGAACGTTTGTAYCTTGTATTG | ACTCACTATCTTCTGTTGATTG | Plasmid |
| IncN_rep | AGTTCACCACCTACTCGCTCCG | CAAGTTCTTCTGTTGGGATTCCG | Plasmid |
| IncN_oriT | TTGGGCTTCATAGTACCC | GTGTGATAGCGTGATTTATGC | Plasmid |
| IncP_oriT | CAGCCTCGCAGAGCAGGAT | CAGCCGGGCAGGATAGGTGAAGT | Plasmid |
| IncQ_oriT | TTCGCGCTCGTTGTTCTTCGAGC | GCCGTTAGGCCAGTTTCTCG | Plasmid |
| IncW_trwAB | AGCGTATGAAGCCCGTGAAGGG | AAAGATAAGCGGCAGGACAATAACG | Plasmid |
| sul2 | TCATCTGCCAAACTCGTCGTTA | GTCAAAGAACGCCGCAATGT | Sulfonamide |
| strB | GCTCGGTCGTGAGAACAATCT | CAATTTCGGTCGCCTGGTAGT | Sulfonamide |
| sulA/folP | CAGGCTCGTAAATTGATAGCAGAAG | CTTTCCTTGCGAATCGCTTT | Sulfonamide |
| sul1 | GCCGATGAGATCAGACGTATTG | CGCATAGCGCTGGGTTTC | Sulfonamide |
| sulIII | CGCGCTCAAGGCAGATG | GGGAATGCCATCTGCCTTG | Sulfonamide |
| tet(36) | AGAATACTCAGCAGAGGTCAGTTCCT | TGGTAGGTCGATAACCCGAAAAT | Tetracycline |
| tet(32) | CCATTACTTCGGACAACGGTAGA | CAATCTCTGTGAGGGCATTTAACA | Tetracycline |
| tetA | CTCACCAGCCTGACCTCGAT | CACGTTGTTATAGAAGCCGCATAG | Tetracycline |
| tetB | AGTGCGCTTTGGATGCTGTA | AGCCCCAGTAGCTCCTGTGA | Tetracycline |
| tetK | CAGCAGTCATTGGAAAATTATCTGATTATA | CCTTGTACTAACCTACCAAAAATCAAAATA | Tetracycline |
| tetQ | CGCCTCAGAAGTAAGTTCATACACTAAG | TCGTTCATGCGGATATTATCAGAAT | Tetracycline |
| tetH | TTTGGGTCATCTTACCAGCATTAA | TTGCGCATTATCATCGACAGA | Tetracycline |
| tetW | ATGAACATTCCCACCGTTATCTTT | ATATCGGCGGAGAGCTTATCC | Tetracycline |

| | | | |
|---|---|---|---|
| tetO | CAACATTAACGGAAAGTTTATTGTATACCA | TTGACGCTCCAAATTCATTGTATC | Tetracycline |
| tetL | ATGGTTGTAGTTGCGCGCTATAT | ATCGCTGGACCGACTCCTT | Tetracycline |
| tetX | AAATTTGTTACCGACACGGAAGTT | CATAGCTGAAAAAATCCAGGACAGTT | Tetracycline |
| tetC | ACTGGTAAGGTAAACGCCATTGTC | ATGCATAAACCAGCCATTGAGTAAG | Tetracycline |
| tetS | TTAAGGACAAACTTTCTGACGACATC | TGTCTCCCATTGTTCTGGTTCA | Tetracycline |
| tetE | TTGGCGCTGTATGCAATGAT | CGACGACCTATGCGATCTGA | Tetracycline |
| tetT | CCATATAGAGGTTCCACCAAATCC | TGACCCTATTGGTAGTGGTTCTATTG | Tetracycline |
| tetD | AATTGCACTGCCTGCATTGC | GACAGATTGCCAGCAGCAGA | Tetracycline |
| tetPB | TGGCAAGACGAGTTTGACTGA | GATCGCTCCACTTCAGCGATAA | Tetracycline |
| tetG_F | TCGCGTTCCTGCTTGCC | CCGCGAGCGACAAACCA | Tetracycline |
| tetR | CCGTCAATGCGCTGATGAC | GCCAATCCATCGACAATCACC | Tetracycline |
| tetPA | GGAAACCTTAGTTCAGTGACTTGG | CCCATTTAACCACGCACTGAA | Tetracycline |
| tetM | GGAGCGATTACAGAATTAGGAAGC | TCCATATGTCCTGGCGTGTC | Tetracycline |
| tetJ | CAGCGCCCATACGCCATTTA | CCTACTTCAGTAGTGTGCCAAGC | Tetracycline |
| Tp614 | GGAAATCAACGGCATCCAGTT | CATCCATGCGCTTTTGTCTCT | Transposase |
| IS613 | AGGTTCGGACTCAATGCAACA | TTCAGCACATACCGCCTTGAT | Transposase |
| tnpA-1 | GCCGCACTGTCGATTTTTATC | GCGGGATCTGCCACTTCTT | Transposase |
| tnpA-2 | CCGATCACGGAAAGCTCAAG | GGCTCGCATGACTTCGAATC | Transposase |
| tnpA-3 | GGGCGGGTCGATTGAAA | GTGGGCGGGATCTGCTT | Transposase |
| tnpA-4 | CATCATCGGACGGACAGAATT | GTCGGAGATGTGGGTGTAGAAAGT | Transposase |
| tnpA-5 | GAAACCGATGCTACAATATCCAATTT | CAGCACCGTTTGCAGTGTAAG | Transposase |
| tnpA-7 | AATTGATGCGGACGGCTTAA | TCACCAAACTGTTTATGGAGTCGTT | Transposase |

| | | | |
|---|---|---|---|
| dfrA1 | GGAATGGCCCTGATATTCCA | AGTCTTGCGTCCAACCAACAG | Trimethoprim |
| dfrA12 | CCTCTACCGAACCGTCACACA | GCGACAGCGTTGAAACAACTAC | Trimethoprim |
| vanC | CCTGCCACAATCGATCGTT | CGGCTTCATTCGGCTTGATA | Vancomycin |
| VanB | TTGTCGGCGAAGTGGATCA | AGCCTTTTTCCGGCTCGTT | Vancomycin |
| vanHD | GTGGCCGATTATACCGTCATG | CGCAGGTCATTCAGGCAAT | Vancomycin |
| vanRA | CCCTTACTCCCACCGAGTTTT | TTCGTCGCCCCATATCTCAT | Vancomycin |
| vanSA | CGCGTCATGCTTTCAAAATTC | TCCGCAGAAAGCTCAATTTGTT | Vancomycin |
| vanWB | CGGACAAAGATACCCCCTATAAAG | AAATAGTAAATTGCTCATCTGGCACAT | Vancomycin |
| vanRB | GCCCTGTCGGATGACGAA | TTACATAGTCGTCTGCCTCTGCAT | Vancomycin |
| vanRC | TGCGGGAAAAACTGAACGA | CCCCCCATACGGTTTTGATTA | Vancomycin |
| vanRD | TTATAATGGCAAGGATGCACTAAAGT | CGTCTACATCCGGAAGCATGA | Vancomycin |
| vanSC | ATCAACTGCGGGAGAAAAGTCT | TCCGCTGTTCCGCTTCTT | Vancomycin |
| vanTE | GTGGTGCCAAGGAAGTTGCT | CGTAGCCACCGCAAAAAAAT | Vancomycin |
| vanTC | ACAGTTGCCGCTGGTGAAG | CGTGGCTGGTCGATCAAAA | Vancomycin |
| vanTG | CGTGTAGCCGTTCCGTTCTT | CGGCATTACAGGTATATCTGGAAA | Vancomycin |
| vanA | GGGCTGTGAGGTCGGTTG | TTCAGTACAATGCGGCCGTTA | Vancomycin |
| vanXA | TCGTTGGGACGCTAAATATGC | GGACGGTAACCGTCCCATA | Vancomycin |

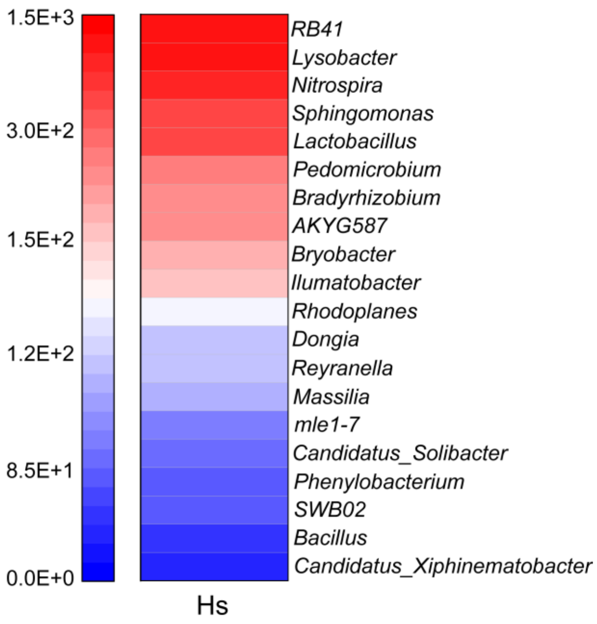


Fig. S1 The top 20 genera in humus soil
